# Examining the Relation Among Subjective Age and Working Memory in Old Age on a High-Frequency Basis Across 7 Days

**DOI:** 10.1093/geroni/igaa057.2011

**Published:** 2020-12-16

**Authors:** Anna Lücke, Jelena Siebert, Oliver Schilling, Denis Gerstorf, Ute Kunzmann, Anna Kornadt, David Weiss, Hans-Werner Wahl

**Affiliations:** 1 Heidelberg University, Heidelberg, Baden-Wurttemberg, Germany; 2 Humboldt University Berlin, Berlin, Berlin, Germany; 3 University of Leipzig, Leipzig, Sachsen, Germany; 4 University of Luxembourg, Esch-sur-Alzette, Diekirch, Luxembourg; 5 Leipzig University, Leipzig, Sachsen, Germany; 6 University of Heidelberg, Heidelberg, Baden-Wurttemberg, Germany

## Abstract

While increasing longitudinal evidence suggests that negative age views accelerate cognitive decline and increase dementia risk, we know little about such co-variance dynamics on a daily basis. We make use of subjective age and working memory performance data obtained six times a day over seven consecutive days as people went about their daily routines from 123 young-old (aged 66-69 years, 47.2% women) and 42 old-old (aged 86-90 years, 55.8% women) adults. Notably, multilevel models revealed considerably-sized short-term intra-individual variation of subjective age and working memory within days and these short-term within-day fluctuations in subjective age and working memory were coupled as expected. Hence, increased subjective age went along with lowered working memory confirming previous research. However, the respective between-day associations appeared reversed. Given this evidence of correlated short-term variability, we also discuss implications of different change dynamics that might explain moment-to-moment versus day-to-day associations between subjective age and working memory.

